# Climate Resilience in Farm Animals: Transcriptomics-Based Alterations in Differentially Expressed Genes and Stress Pathways

**DOI:** 10.3390/biotech13040049

**Published:** 2024-11-15

**Authors:** Chikamagalore Gopalakrishna Shashank, Veerasamy Sejian, Mullakkalparambil Velayudhan Silpa, Chinnasamy Devaraj, Aradotlu Parameshwarappa Madhusoodan, Ebenezer Binuni Rebez, Gajendirane Kalaignazhal, Artabandhu Sahoo, Frank Rowland Dunshea

**Affiliations:** 1 Centre for Climate Resilient Animal Adaptation Studies, ICAR-National Institute of Animal Nutrition and Physiology, Adugodi, Bangalore 560030, India; shanko009@gmail.com (C.G.S.); drcdeva@gmail.com (C.D.); binunirebez.e@gmail.com (E.B.R.); sahooarta1@gmail.com (A.S.); 2 Rajiv Gandhi Institute of Veterinary Education and Research, Kurumbapet 605009, India; mv.silpa@gmail.com; 3 Temperate Animal Husbandry Division, ICAR-Indian Veterinary Research Institute, Mukteswar Campus, Nainital 263138, India; madhu.jnvet.918@gmail.com; 4 Department of Animal Breeding and Genetics, College of Veterinary Science and Animal Husbandry, Odisha University of Agriculture and Technology, Bhubaneshwar 751003, India; gnazhal99@gmail.com; 5 School of Agriculture, Food and Ecosystem Sciences, Faculty of Science, The University of Melbourne, Parkville, Melbourne, VIC 3010, Australia; 6 Faculty of Biological Science, The University of Leeds, Leeds LS2 9JT, UK

**Keywords:** climate change, transcriptomics, livestock, adaptation, biomarkers

## Abstract

The livestock sector, essential for maintaining food supply and security, encounters numerous obstacles as a result of climate change. Rising global populations exacerbate competition for natural resources, affecting feed quality and availability, heightening livestock disease risks, increasing heat stress, and contributing to biodiversity loss. Although various management and dietary interventions exist to alleviate these impacts, they often offer only short-lived solutions. We must take a more comprehensive approach to understanding how animals adapt to and endure their environments. One such approach is quantifying transcriptomes under different environments, which can uncover underlying pathways essential for livestock adaptation. This review explores the progress and techniques in studies that apply gene expression analysis to livestock production systems, focusing on their adaptation to climate change. We also attempt to identify various biomarkers and transcriptomic differences between species and pure/crossbred animals. Looking ahead, integrating emerging technologies such as spatialomics could further accelerate genetic improvements, enabling more thermoresilient and productive livestock in response to future climate fluctuations. Ultimately, insights from these studies will help optimize livestock production systems by identifying thermoresilient/desired animals for use in precise breeding programs to counter climate change.

## 1. Introduction

Extreme climatic shifts are anticipated, with global warming approaching 1.5 °C by 2040 and average surface temperature predictions ranging from 1.88 to 4.08 °C (depending on the models used and the assumptions made) by 2100 [1]. The emission of greenhouse gases (GHGs) causes atmospheric warming, contributing to global climate change [2]. With the current and anticipated surge in global population, climate change is expected to impact livestock production in various ways, including intensified competition for natural resources, reduced feed quality and availability, increased disease prevalence, heat stress, and loss of biodiversity. These challenges arise as the livestock industry strives to meet a projected 100% increase in demand for animal products by the mid-21st century [3].

The livestock sector is essential for sustaining food supply and security. Products from livestock, such as meat, milk, and eggs, contribute approximately 15% of global per capita calorie intake and 31% of protein intake, with some variation across different regions [4]. Agriculture is a source of income for over 844 million people worldwide, with over 40% of the value from the livestock sector [5]. The effect of climate change can be either positive or negative depending on region, species, and production type in livestock [6]. For example, an increase in the yield of crops and pasture due to the increasing climate changes is expected in Central Europe, which could be beneficial for livestock feeding [7]; on the other hand, regions like southern Australia [8] and southern South Africa, which rely on winter rainfall, may decline [9]. If particularly concentrating on tropical climate livestock production, serious concerns elevate the importance of thermoresilience against future global warming.

Particularly in tropical countries, more research is necessary related to strategies such as breeding livestock for adaptability rather than exclusively focusing on high productivity. Although a variety of management and dietary strategies are available to mitigate the effects of heat stress on livestock, these approaches may offer only short-term relief. Thus, a more thorough understanding of the genetic diversity and molecular mechanisms that drive thermotolerance and natural resilience is essential. To successfully mitigate the negative effects of climate change and maximize production, the best adaptation strategies should integrate technological, behavioral, management, and policy choices. Narrowing down to technological aspects, several molecular approaches, including quantitative polymerase chain reaction (qPCR), microarrays, and RNA sequencing (RNA-seq), can be employed to investigate the transcriptome of an individual. RNA-seq has introduced a novel way of measuring gene expression across the entire transcriptome, including in non-model animals for which no prior sequencing information is available [10]. This comprehensive review focuses on applying RNA-seq approaches to comprehend adaptation in various farm animals, followed by variations in transcripts in response to exposure to heat stress. The review concludes by identifying traditional biomarkers that may inform future breeding strategies across different farm animal species.

## 2. Economic Consequences of Heat Stress on Livestock Production

When environmental conditions defy the animal’s thermoregulatory mechanisms, the state is known as heat stress. Heat stress is a multibillion-dollar problem for the global livestock system. The impacts of heat stress include reduced efficiency, increased vulnerability to diseases, compromised animal welfare, and reduced fertility [11]. The general response to heat stress and the point at which production losses activate vary widely between animals. Various climate change assessment systems predict an increase in temperature from 1.1 °C to 6.4 °C by the end of this century [12]. Globally, numerous livestock species are likely to be impacted negatively due to this fluctuation in temperature.

Assessing the influence of heat stress on economic losses can be accounted for in three ways: (1) Decreased productivity (growth, milk, meat, and wool), (2) increased mortality, and (3) reduced reproductive efficiency. Metabolic heat production is greater in high-producing dairy cows compared to low-producing cows. Therefore, cows producing large amounts of milk are more susceptible to heat stress. For instance, due to heat stress, the United States livestock industry faced an annual economic loss between USD 1.69 and 2.36 billion, of which a major loss was found in the dairy and beef industry [13]. According to a study, the average milk production per cow in the United States could decline by 11.6 per cent on average by the mid-twentieth century and by 20.8 per cent on average by the late-twentieth century due to heat stress, resulting in massive revenue losses for the dairy industry [14]. A study finding projected an economic loss of roughly 16.5 million (AUD) to Australian feedlots due to heat stress [15]. In Canada, it is predicted that milk fat and protein content would decrease due to heat stress, thus resulting in a loss of CAD 5.34 to 7.07 ·hL^−1^ [16]. Most of the estimates analyzed are outdated and do not represent the current economic impact of heat stress. In addition, heat stress on cattle production systems may likely result in greater economic losses than these figures indicate.

The above-mentioned are economic losses due to the direct effect of heat stress on livestock; considering the indirect effects, these include impact on fodder crops, scarcity of water, occurrences of diseases in livestock and fodder, veterinary costs linked to the increased incidence of associated production diseases, and many more. Finally, this leads to huge economic losses to farmers and to nations.

Livestock production is among the most climate-sensitive economic sectors, making vulnerable communities especially susceptible to heat stress effects. Across many regions, livestock systems are rapidly evolving in response to factors such as population growth, rising demand for animal products linked to income growth, and urbanization. The impacts of heat stress are felt most acutely by low-income populations that depend heavily on natural resources for their livelihoods. Managing livestock under heat stress requires adaptation strategies to reduce production losses, with options ranging from low- to high-cost interventions. Low-cost adaptations might include reducing overcrowding and maximizing shade availability, while moderate-cost strategies may involve installing sprinklers to cool animal shelters and enhancing ventilation. High-cost measures could include constructing climate-controlled animal facilities and providing high-quality feed. However, livestock owners often lack the financial resources and technological capacity needed to implement these adaptations to counter projected temperature increases and related climatic changes. For rural populations, losing livestock assets can lead to chronic poverty, with enduring consequences for their livelihoods.

## 3. Significance of Understanding the Molecular Mechanisms of Livestock Adaptation

Understanding cellular responses to climate change might provide clues to unravel the mystery behind the complexities enabling cells to adapt to the changing environment. The impacts of environmental stress on molecular mechanisms are still in their infancy, but they may someday explain the diversification of animal genetic resources.

Reductions in the productive performance of cattle due to heat stress are attributable in great proportion to the homeokinetic shifts animals endure to maintain an ideal core body temperature. However, cattle adapted to hotter climates appear to have acquired genes that shield cells from the damaging effects of heat [17]. The practical utility of the previous statement can be that “rather than relying on crossbreeding alone to utilize the zebu genotype for livestock production in hot climates, we can instead introduce the zebu genes that confer thermo tolerance into European breeds while excluding the undesirable genes” [17]. For instance, a slick mutation in the prolactin receptor gene (*PRLR*) identified in Senepol cattle has been incorporated into other dairy breeds in regions like Puerto Rico, Florida, and New Zealand. Studies later confirmed that Florida Holsteins carrying the slick phenotype showed lower rectal and vaginal temperatures and reduced respiration rates under heat stress compared to Holsteins lacking this phenotype [18]. Similarly, if other mutations conferring thermotolerance are found, either at the whole-animal or cellular level, it will open up new possibilities for employing genetic interventions to mitigate the effects of heat stress. To quote further examples, researchers working on genome-wide selection for thermotolerance used a heat shock gene marker to identify a bull suitable for use in the breeding program [19]. Additionally, exposure of an animal to heat stress has been shown to affect the expression of genes outside of the heat shock protein family, such as the *ATP1A1* gene (Jersey crossbred cows) [20] and the *ATP1B2* gene (Chinese Holstein cows) [21], which are relevant to thermotolerance. These SNPs can potentially serve as markers for the early development of thermotolerance in animals. In addition, a bull with thermotolerance can be used in breeding programs to produce animals with better thermoresilience. 

To illustrate the application of our current knowledge of cellular and molecular pathways in livestock, Canadian dairy herds saw a 6% increase in milk production between 2005 and 2012, despite an 11% reduction in the number of dairy cows [22]. A significant gain in milk output and productivity can be attributed to both ongoing genetic selection and advances in the biology of lactation and biosynthesis of milk. Through different sequencing technologies like metagenomics, transcriptomics, proteomics, and metabolomics, specific genes and gene variants that play a significant role in carrying milk production traits can be identified, which can increase milk production. A missense mutation in the *DGAT1* gene on chromosome 14 has been found to profoundly impact milk fat and composition in dairy cattle [23]. Further, on chromosomes 6 and 20, the *ABCG2* and *GHR* genes have been found to have significant effects on milk yield and composition [24]. Thus, advancements in molecular techniques bring us closer to uncovering the fundamental mechanisms underlying livestock adaptation to diverse environments. However, policymakers play a crucial role in recognizing the significance of the breeding policies derived through countless hours of research and knowledge invested by researchers in propagating the present knowledge towards a sustainable future.

## 4. Transcriptomics-Based Approach for Studying Molecular Mechanisms of Livestock Adaptation

A delicate equilibrium between an organism and its surroundings is constantly reviewed through the interpretation of environmental cues and the subsequent “adjustments” to the transcriptome that modify the animal’s physiology. Gaining insight into how animals adapt and thrive in their environments often calls for straightforward approaches. One approach can be quantifying transcriptomes under different environments, which can uncover underlying pathways essential for livestock adaptation. Accessibility and affordability continue to promote the rapid expansion of transcriptomics as a science. Transcriptomics is no longer restricted to a select few [25].

Various transcriptomics studies have been conducted to understand the adaptation of livestock to various environments. Namely, a study was carried out on transcriptomic variation in peripheral blood mononuclear cells (PBMCs) in native Ladakhi cattle, which dwell at altitudes of around 3500–5500 m above sea level [26]. The researchers identified genes like *ITPRI*, *INHBC*, *HECA*, *GPR171*, *ABI3*, and *HIF-1α* that were up-regulated in response to hypoxia and stress response at high altitude. Furthermore, the authors validated that genes like *HIF-1*, *EPAS-1*, *VEGFA*, *NOS2*, and *GLUT-1/SLC2A*1 in Ladakhi cattle play a major role in high-altitude adaptation. Similarly, a study was conducted revealing cattle’s adaptive capacity to sub-arctic conditions [27]. Authors identified novel genes like *BHLHE40* and *PRKCG* linked to circadian rhythms. The importance of these genes in Yakutian cattle was to metabolically prepare for food scarcity during long winters [28]. Further, a study focused on understanding hepatic transcriptomic adaptation during the transition period in dairy cows [29]. The authors established that the liver’s primary adaptation to the early postpartum period in cows is metabolic, focusing on lipid metabolism, with fatty acid oxidation and mitochondrial activity playing key roles. Further bioinformatics analyses showed that numerous signaling pathways, most notably peroxisome proliferator-activated receptor (PPAR) and adipocytokine signaling, were crucial in the activation of metabolism, particularly lipid, glucose, and amino acid metabolism. In other breeds, the adaptation of cashmere goats to cold in the temperate Himalayan region [30], the drought resistance capacity of camels to the harsh desert environment [31], higher altitude adaptation of yaks [32], hypoxic adaptation of Tibetan chickens [33,34,35,36], and heat resistance mechanisms in buffaloes are further adaptive examples.

Transcriptomics technology could generate helpful information to understand the adaptation mechanisms of resilient breeds and/or species. Its application in tropical animal production may act as a key to the development of adaptation and mitigation strategies for a changing climate. Additionally, transcriptomics offers actionable insights that allow breeders and farmers to integrate molecular findings into breeding, management, and facility adaptations. For example, recent studies have identified several genes essential for thermotolerance, including *HSP70* and *HSP90*, which help cells withstand heat by preventing protein denaturation. Dangi et al. [37] demonstrated that upregulating these genes improves resilience in goats, providing a direct breeding target for thermotolerance. In addition, as mentioned previously, the “slick” prolactin receptor gene (*PRLR*) mutation first observed in Senepol cattle has been integrated into Holstein dairy cattle to improve thermotolerance. Studies show these Holsteins maintain lower core body temperatures and respiration rates under heat stress, enhancing survival and productivity in tropical environments [18]. This selection process is further streamlined by marker-assisted selection (MAS), which enables breeders to focus on specific resilience markers. For example, the *DGAT1* and *ABCG2* genes, known for their roles in milk composition and thermotolerance, have been integrated into MAS for dairy cattle. Grisart et al. [23] demonstrated that *DGAT1* markers significantly increased milk yield and improved resilience to heat stress, highlighting MAS’s effectiveness in selecting for both productivity and climate resilience. Emerging technologies like CRISPR-Cas9 also allow precise modifications of genes associated with thermotolerance, such as *ATP1A1* in dairy cattle. Research has shown that modifying this gene improves cellular resilience to heat, indicating CRISPR’s potential to produce climate-resilient livestock that can sustain productivity under rising temperatures [38].

Supportive policies encouraging the use of transcriptomic data in breeding programs, such as subsidies for thermotolerant genetic markers, would accelerate climate resilience in livestock. Policies that promote MAS, CRISPR, and PLF systems can make sustainable livestock production more accessible and ensure that the industry is equipped to address the challenges posed by climate change. By integrating transcriptomics into breeding, management, and environmental adaptations, the livestock industry is poised to build a robust, climate-adaptive sector capable of meeting future demands in a warming world.

## 5. Species Differences in Significantly Altered Transcripts in Heat-Stressed Farm Animals

Exploring the molecular mechanisms in animals under heat stress could reveal targets for developing new strategies to enhance animal immunity. In a transcriptome analysis of 10 Holstein bull calves, 8567 genes were differentially regulated in response to heat stress, with 465 genes significantly up-regulated (≥2-fold, *p*  <  0.05) and 49 genes significantly down-regulated (≤2-fold, *p*  <  0.05). [39]. Key pathways enriched in response to heat stress include chaperones, co-chaperones, cellular response to heat stress, immune response, apoptosis, Toll-like receptor signaling, Pi3K/AKT activation, protein processing in the endoplasmic reticulum, interferon signaling, estrogen signaling, and MAPK signaling. The authors identified ten highly up-regulated genes following heat exposure, such as heat shock 70 kDa protein 1A (HSPA1A), heat shock 105 kDa/110 kDa protein 1 (HSPH1), heat shock 70 kDa protein 8 (HSPA8), DnaJ (Hsp40) homolog subfamily A, member 1 (DNAJA1), and CDC-like kinase 1 (CDK1). Most of these genes are associated with metabolic processes, catalytic activity, transcription factor activity, enzyme regulation, protein binding, apoptosis, stimulus response, cellular processes, and immune functions. Additionally, heat-responsive genes and pathways were identified in heat-stressed buffalo mammary epithelial cells (MECs) [40]. In heat-stressed MECs, several genes from the heat shock family were elevated, including *HSPA6*, *HSPB8*, *DNAJB2*, and *HSPA1A*. Beyond heat shock proteins, other elevated genes included *BOLA*, *MRPL55*, *PFKFB3*, *PSMC2*, *ENDODD1*, *ARID5A*, and *SENP3*. Transcriptome data indicate that these heat-responsive genes are involved in various functional roles, such as immune response, chaperone activity, cell proliferation, and metabolism. Key pathways identified include the electron transport chain, cytochrome P450, apoptosis, IL2 signaling, MAPK, fatty acid synthase, stress-induced HSP regulation, delta Notch signaling, HSP70-modulated apoptosis, EGFR1 signaling, cytokine and inflammatory response, nuclear receptors, oxidative stress, TNF-alpha/NF-kB, and GPCR pathways. This research sheds light on the molecular foundation of the heat stress response in bovine species like cattle and buffalo. To deepen the understanding of post-transcriptional regulation under heat stress in various livestock, examining genetic variations in miRNA target sites and miRNA profiling under stress conditions could be valuable.

A study on sheep was conducted to investigate the genes and pathways that play regulatory roles. The authors observed that stress-related genes, such as *Rap1*, *MAPK*, and *PI3K-Akt*, were significantly enriched in the functional enrichment analysis of differentially expressed genes between heat-stressed and control sheep. Additionally, genes linked to cAMP signaling pathways, including *NPR1*, *ANGPT2*, and *SLC12A5*, were involved in fat regulation mechanisms. For sheep on exposure to heat stress, the mentioned stress-related signaling pathways are capable of forming a complex cascade in response, thereby mitigating the damage caused by such stress. Further, sheep had to mobilize a great deal of energy, which increased catabolism and decreased anabolism proportionally. Therefore, the mobilization of fat in heat-stressed sheep could aid in their adaptation [41]. Similarly, in transcriptomic studies involving chicken, researchers identified 245 differentially expressed genes, of which 230 genes were up-regulated and 15 were down-regulated. *HSP*s, *MYLK2*, and *BDKRB1* genes were identified as key genes during heat stress as up-regulated differentially expressed genes. Further, the KEGG study revealed connections to the ATP metabolic process, the MAPK signaling pathway, and the calcium signaling pathways, all of which are associated with protein processing and synthesis [42].

Heat stress also affects the fertility of animals. The reproductive decline in boars caused by heat stress is a growing danger to the industry’s bottom line. The US alone witnessed a loss of USD one billion per year in recent years [43]. To understand and address the genes and metabolic mechanisms involved with heat tolerance in pigs, a study identified *SLC16A2*, *MARCHF1*, *TNFAIP6*, *RXFP2*, and *IL15* genes contributing to the heat resistance and immune function activation in pigs when subjected to heat stress [44]. Additionally, distinct co-expression patterns during heat stress were observed between heat-tolerant and heat-susceptible groups. According to the authors, the above-mentioned genes and a few others in the literature can help in the development of biomarkers for heat tolerance ability and provide new insight into the biological mechanisms that underlie heat tolerance in pigs. The impact of heat stress on fertility and embryo development has been the subject of several in vivo and in vitro studies. A study suggested five common genes, namely, *E2F8*, *GATAD2B*, *BHLHE41*, *FBXO44*, and *RAB39B*, and various pathways such as glucocorticoid biosynthesis, apoptosis signaling, and HIPPO signaling in GV oocytes, and Oct4 pluripotency, Wnt/beta-catenin signaling, and melatonin degradation, to be significantly affected by heat stress during the embryo’s development through transcriptomic studies [45]. Similarly, during summer, the expressions of various genes, such as *HSP90AA1*, *HSPA1AB*, *NRF1*, *POLG2*, *GDF9*, *BMP15*, and *FGF16*, were positively associated with the development outcome into blastocysts [46]. Table 1 summarizes the transcriptomics-aided identification of genes associated with heat stress response in farm animals.

The main purpose of analyzing and understanding the transcripts in livestock, especially on exposure to heat stress, can be an effective tool to ameliorate the impacts of climate change, particularly global warming, on livestock and associated sectors. The genes and pathways responsible for thermotolerance in livestock can be exploited and utilized in technologies like CRISPR-Cas9 to edit the complete genome system to produce animals that can sustain the upcoming consequences of heat stress without any compromise to global food security.

## 6. Differences in Heat Stress-Mediated Significantly Altered Transcripts Between Indigenous and Crossbred/Purebred Animals

Apart from having significance in understanding the molecular mechanism associated with livestock adaptation, RNA-seq technology also aids in comparing the transcriptome profile among breeds [47]. Such breed-wise comparisons provide insight into the high-resolution screening of variations in differential gene expressions, thereby providing a deeper understanding of the functional levels that lead to phenotypic differentiation among the livestock breeds [47]. There are fewer reports on adopting transcriptomics to assess the impact of heat stress in livestock and compare the adaptive differences among livestock breeds. While most studies aimed to understand the transcriptomic profile of a single breed subjected to heat stress (Murciano-Granadina goats [48]; Holstein cows [49]), very limited studies focused on assessing the breed differences [50,51], which, however, is vital to understand the differences in heat stress response and adaptive potential among livestock breeds.

In a recent report, the transcriptomic profile of two Indian cattle breeds, Tharparkar (Indigenous breed) and Vrindhavani (Crossbred), were assessed to understand the system biology governing thermotolerance in indigenous cattle [51]. Both cattle breeds were exposed to acute heat stress, and their PBMC transcriptomic profiles were analyzed. The study reported a notable difference in the number of differentially expressed genes (DEGs) between the two breeds, with 6042 DEGs identified in crossbred cattle compared to 4718 DEGs in the indigenous breed. An interesting finding was the contrasting patterns in gene expression between the breeds: approximately 18.5% of the up-regulated DEGs in the crossbred Vrindavani cattle were down-regulated in the indigenous Tharparkar cattle, and similarly, 17.5% of the up-regulated DEGs in Tharparkar cattle were down-regulated in the crossbred cattle. This study highlighted a significant dysregulation in the biological systems between the breeds and noted differences in the expressions of several heat stress-associated genes, suggesting a stronger heat stress resilience in Tharparkar cattle.

Another study examined the transcriptome response to heat stress in two indigenous chicken ecotypes in Kenya [50]. The two indigenous chicken ecotypes were selected from distinct regions: the lowland (hot and humid Mombasa) and the highland (cooler Naivasha). In addition to examining the differences in response to acute and chronic heat stress, the authors analyzed the variations in heat stress responses between cardiac and skeletal muscle tissues using transcriptomic analysis. The study results revealed a distinct difference in the differentially expressed genes (DEGs) between the lowland and highland chickens. A total of 351 (skeletal muscle) and 384 (cardiac muscle) genes were significantly differentially expressed in lowland chicken due to acute heat stress, while the same for highland chicken was 322 (skeletal muscle) and 184 (cardiac muscle) DEGs. Likewise, differences in DEGs between the two chicken ecotypes were also observed under chronic heat stress. The transcriptome profiling of skeletal and cardiac muscle of lowland chicken under chronic heat stress revealed 142 and 172 DEGs, respectively, to be significantly altered. The significant DEGs altered due to chronic heat stress in highland chicken’s skeletal and cardiac muscles were 180 and 170, respectively. Further studies assessing the types of DEGs altered, the differences at the functional level, and comparative assessment between the chicken ecotypes and types of heat stress reflected differences in the acclimatization rate of lowland and highland chickens. Therefore, a stronger and deeper insight into the heat stress response between two chicken ecotypes was obtained using transcriptomics. To add, recent studies have delved into the innate differences between chicken breeds via single-cell transcriptome analyses [52], shedding light on the genetic mechanisms underlying the adaption, growth, and development of different breeds. To further understand the response to heat stress of chickens, more in-depth research using single-cell transcriptome is warranted.

A transcriptomics approach was also adopted to unravel the underlying heat tolerance mechanism in Turpan black sheep (known for its excellent thermotolerance in China) using heat-sensitive Kazakh sheep as the control [53]. Transcriptome profiling of pituitary, ovarian, and hepatic tissues revealed 32, 49, and 69 DEGs, respectively, to be up-regulated due to heat stress in Turpan black sheep compared to Kazakh sheep. Likewise, 39, 60, and 145 DEGs were down-regulated due to heat stress in the pituitary, ovarian, and hepatic tissues of Turpan black sheep in comparison to Kazakh sheep, respectively. The results obtained from the study provided insight into understanding the adaptability of Turpan black sheep to harsh environments without compromising its fecundity, thereby aiding towards selection for heat resistance.

Therefore, transcriptomic analysis can be considered an effective tool that has the potential to provide a deeper knowledge of the molecular mechanisms governing response to heat stress among livestock breeds. Its added application in screening for thermotolerant breeds that can be used for future breeding programs.

## 7. Heat Stress-Associated Changes in Clusters of Orthologous Gene (COG) Level Functions in Farm Animals

Recent advances in the field of biotechnology, particularly genome sequencing, have resulted in a rapid enrichment of protein databases; most of the proteins’ functional roles are not yet defined. In this scenario, computational biology plays a major role in striving to find the maximum possible information from the genome sequences, thereby categorizing them based on their homologous relationships and predicting their possible biochemical and physiological functions, three-dimensional structures, and evolutionary origins [54].

Orthologs are genes of different organisms that evolved from a common ancestral gene of the targeted species, and generally, orthologs preserve the same function during evolution. Accurate identification and characterization of orthologs is a critical step for comparative genomic studies and prediction of gene function in newly sequenced genomes [55]. The Clusters of Orthologous Groups of Proteins (COGs) database is an attempt to sort proteins from complete genome sequences based on the orthology concept [56]. COG analysis is commonly used to classify the gene functions based on the homologous classification of gene products using the COG database [54,57].

A study adopted COG analysis to assess the function of the assembled unigenes. Based on the COG analysis, they grouped unigenes into 24 COG functional categories [58]. In the COG categories, the signal transduction mechanisms cluster was the largest, comprising 15.50% of the heat-treated Muscovy duck groups. This high cluster abundance suggests that these genes play an essential regulatory role in senescence and stress responses. Twenty-four categories of clusters were identified via COG analysis of all the proteins identified from the bovine mammary epithelial cells exposed to heat stress conditions. Among the clusters, the general function prediction cluster was the largest group, followed by the lipid transport and metabolism, amino acid transport and metabolism, and carbohydrate transport and metabolism, which were the larger groups [59]. They also identified 95 proteins with COG categories, which were related to lipid metabolism.

A study utilized the eggNOG database to predict protein domains by analyzing the distribution of clusters of orthologous groups in rumen samples from Jersey and Holstein cows [60]. The study found that certain protein domains, such as those associated with energy production and conversion, were more abundant in the control group compared to the heat-stressed group, where heat stress reduced the relative proportion of these genes in Jersey cows. Additionally, COG analysis showed an increased abundance of genes related to defense mechanisms, lipid transport and metabolism, coenzyme transport and metabolism, cell cycle control, cell division, and chromosome maintenance in heat-stressed Holstein cows, while these gene abundances remained unaffected by heat stress in Jersey cows. Heat stress also lowered the relative abundance of genes related to cell motility and extracellular structures in Holstein cows but not in Jersey cows [60].

The study results showed no significant difference in the abundance levels of most COGs between the control and heat-stressed groups in Osmanabadi and Malabari goat breeds [61]. In contrast, the Salem black goat breed exhibited a decrease in the abundance of most COGs in the heat-stressed group compared to the control. COG analysis led to the conclusion that heat stress did not impact the relative abundance of predicted functional rumen metagenome pathways in the Osmanabadi and Malabari breeds. However, the lower abundance of these pathways in heat-stressed Salem black goats suggested this breed’s thermal tolerance capacity.

A study used PICRUSt software to predict gene functions in Tibetan sheep across different seasons, identifying 25 COG gene families in the 16S rRNA gene sequencing data from rumen samples. Of these, 13 gene families showed significant differences between the cold and warm seasons [62]. The study observed a reduction in the abundance of COG gene categories related to carbohydrate transport and metabolism, alongside an increase in COG genes linked to energy production and conversion, meeting the energy demands of sheep during the cold season. This pattern was consistent with findings from the KEGG pathway analysis, where COG gene families involved in sugar biosynthesis and metabolism also increased during colder conditions.

A differential COG analysis of rumen microbiome sequences data revealed about 25 COG protein functional gene categories during April and August. Nine differential COG functional gene families identified were associated with metabolic pathways, which is around 13.67%. The abundance of COG genes related to the amino acid transport and metabolism pathway (7.63%) was significantly increased in April compared to December [63]. The COG functional analysis of rumen microbiota identified the functional classification of COG genes broadly within the area of metabolism, mainly amino acid, carbohydrate, and lipid metabolism and transport. The relative abundance of COG gene families associated with amino acid transport and metabolism was increased by about 51.6%, while there was a 43.8% abundance of COG gene families associated with carbohydrate transport and metabolism. The COG families related to lipid transport and metabolism showed significant increases in abundance, around 50% [64].

## 8. Heat Stress-Associated Changes in Transcriptomics-Based Stress Pathways at Different KEGG Levels in Ruminants

**Figure 1:**
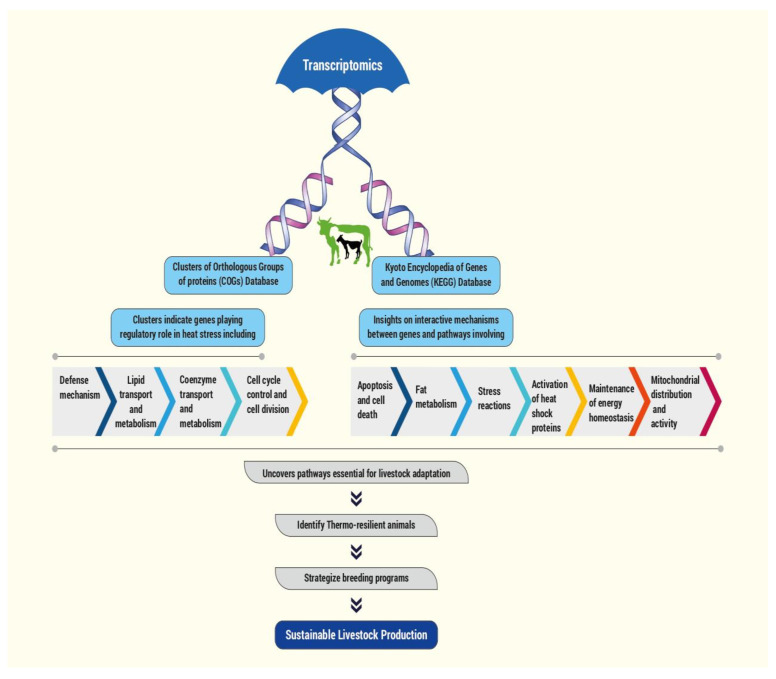
Application of transcriptomic data analysis using Clusters of Orthologous Groups of proteins (COGs) and Kyoto Encyclopedia of Genes and Genomes (KEGG) database in identifying changes associated with heat stress.

Transcriptomic analysis was developed based on RNA sequencing (RNA-seq), which can generate a set of expressed genes in a particular tissue, paving the way for determining differentially expressed genes [65]. It can be widely explored for determining differentially expressed genes associated with heat stress. Heat stress transcriptomic data analysis with the KEGG database could pave the way for identifying superior thermotolerant and productive ruminants for future genetic selection. Transcriptomics data facilitate the detection of major genes and pathways involved in heat stress regulation, whereas the KEGG database provides insight into the interactive mechanisms between genes and pathways. This aids in breeding genetically superior thermotolerant animals. Figure 1 describes the application of transcriptomic data analysis using Clusters of Orthologous Groups of proteins (COGs) and Kyoto Encyclopedia of Genes and Genomes (KEGG) database in identifying changes associated with heat stress. 

Heat stress causes abnormalities in cell function by disrupting transcription factor mechanisms, which in turn affects protein synthesis and leads to structural defects in proteins [66,67,68]. Transcriptome analysis of genes and pathways responsive to heat stress in Holstein calves identified 31 transcription factors, 13 transcriptional cofactors, and 6 chromatin remodeling factors that were differentially expressed. Transcription factors involved in cell cycle regulation, such as *ARID4A*, *ELF4*, *HBP1*, *TFCP2*, and *TFDP1*, were up-regulated, whereas *SPDEF* was down-regulated [37]. A separate study examined the effects of heat stress on the transcriptome profiles of bovine peripheral white blood cells and milk somatic cells. In peripheral white blood cells, genes associated with fatty acid beta-oxidation, oxidation–reduction, potassium ion transport, and digestion were down-regulated, while genes involved in lipopolysaccharide response, nucleic acid binding, and insulin-like growth factor signaling were up-regulated. The differentially expressed genes were linked to 11 biological pathways, including calcium signaling, ketone body synthesis and degradation, and butanoate metabolism. In milk somatic cells, genes involved in growth factor activity, DNA binding, lipid biosynthesis, and endopeptidase inhibition were down-regulated, whereas those related to lipid metabolism, apoptosis, and stress response were up-regulated. The differentially expressed genes were significantly associated with four biological pathways: Wnt signaling, antigen processing and presentation, and type I diabetes. In both cell types, genes linked to carbohydrate metabolism, oxidative stress, and proteolysis were down-regulated, while those related to oxidation–reduction, apoptosis, and stress response were up-regulated [69].

The impact of summer heat stress on circulating microRNA expression in lactating Holstein cows was analyzed using the KEGG pathway database. This analysis identified 18 statistically significant pathways related to the heat stress response, including the FoxO signaling pathway, regulation of the actin cytoskeleton, peroxisome function, Rap1 signaling pathway, TNF signaling pathway, and chemokine signaling pathway [70]. These pathways are involved in activating heat shock proteins, maintaining energy homeostasis and mitochondrial distribution and activity [71,72,73] heat stress exposed the alterations in cell response to stress, cell death, and branching morphogenesis. It indicated aberrant cellular processes, impaired mammary development, and extended inflammation and immune response [74].

KEGG analysis of transcriptome data of hypothalamus tissue of heat-stressed Hu sheep was undertaken at the levels of cellular processes, metabolism, genetic information processing, environmental information processing, and organismal systems. It revealed that the gene expression profile was highly altered in environmental information processing and organismal systems. Several pathways, such as the calcium and MAPK signaling pathways, were involved in heat stress regulation in Hu sheep. In the metabolism process, genes involved in oxidative phosphorylation were altered. Genetic information processes such as replication, folding, degradation, repair, transcription, translation, and sorting were also affected. Heat stress also alters the genes regulating nervous, endocrine, circulatory, excretory, immune, digestive, and sensory systems at the organismal level [75]. KEGG pathway analysis of transcriptome data on the heat stress response in sheep showed that differentially expressed genes were significantly enriched in 51 pathways associated with fat metabolism, stress responses, and apoptosis [41].

In heat-stressed water buffaloes, transcriptome analysis of blood cells identified differentially expressed genes between heat-tolerant and non-heat-tolerant buffaloes through miRNA-seq analysis. The study investigated the genetic mechanisms of heat tolerance in buffaloes using an mRNA–miRNA interaction network to identify hub genes associated with heat tolerance [34].

The transcriptomic analysis of blood cells in chronically heat-stressed lactating dairy goats revealed that 31 biological pathways were impacted, with 3 pathways up-regulated and 28 down-regulated. The up-regulated pathways were associated with apoptosis and cell death, while the down-regulated pathways were primarily related to immune cell proliferation and migration, lipid metabolism, and tissue repair [48].

## 9. Classical Molecular Biomarkers for Heat Resilience in Farm Animals

Before diving into this section, let us first define “molecular marker.” Any observable or measurable phenotype, or its genetic foundation used to assess phenotypic variability, can serve as a genetic marker. A molecular marker specifically refers to a DNA variation between individuals that is associated with certain traits [76]. These variations include insertions, deletions, translocations, duplications, and point mutations. Their unique biological properties at any given time make it possible to detect and quantify them in any bodily fluid or tissue [77]. Several molecular markers in farm animals identified for thermoresilience are mentioned below in Table 2.

## 10. Integrating Omics Approaches for Climate Resilience in Livestock

Integrating transcriptomics with other omics approaches, such as genomics, proteomics, epigenomics, and metabolomics, provides a multi-dimensional framework for enhancing climate resilience in livestock by revealing deeper insights into the molecular mechanisms governing heat tolerance potential [96]. These technologies are considered to be effective tools, especially in the tropical regions, when used in conjunction with advanced molecular and breeding methods [97]. Each omics technology offers a distinct view on the animal’s adaptation mechanism, thereby providing a comprehensive and integrated knowledge on the intricate biological mechanisms underpinning the adaptive processes to the changing climate.

Genomic approaches enable the detection of genetic variations that influence an animal’s ability to withstand heat stress, providing valuable insights into the molecular basis of thermal adaptation [98]. Among the omics approaches, genomics in particular has the potential to improve precision and efficacy of conventional breeding and advanced breeding approaches by enhancing consistency and predictability. Proteomics complements transcriptomics by offering functional insights into how genes translate into biological responses. By studying alterations at the protein level in processes such as energy metabolism, protein stabilization, oxidative stress, and signaling, proteomics provides insights into the molecular mechanisms of heat tolerance. Similarly, metabolomics that studies the metabolic changes helps in understanding the molecular responses to heat stress [99]. These approaches aid in the identification of biomarkers linked to thermotolerance, that serve as valuable tool for improving climate resilience potential.

However, the successful application of omics technology requires proper handling of omics data as well as extensive statistical and bioinformatics understanding [100]. Hence, a more robust study improving data integration methods and analytical tools will ensure more effective and accurate applications in livestock production. In conclusion, a comprehensive approach combining omics technologies can significantly enhance the breeding programs through the incorporation of the precise thermotolerant biomarkers identified through these technologies. This aids in improving the thermotolerance potential in animals to withstand the harsh climatic conditions without losing their production potential. Therefore, such an effort will help in sustaining livestock production to feed the growing human population in the future.

## 11. Conclusions

Due to the complexity of climate change, no single method alone is sufficient for fully understanding livestock adaptation. Modifications in animal management practices, such as reproduction, nutrition, and health care, are necessary but may only partially bridge the gap toward sustainable livestock production in a changing climate. A more lasting solution lies in genomic selection and effective breeding strategies. However, implementing these strategies requires a thorough understanding of the molecular biomarkers within the population. Transcriptomics is a valuable tool in this regard. By conducting gene expression studies, particularly global mRNA expression profiling, we can gain insights into the mechanisms that regulate livestock phenotypes.

Biological pathways and their regulation are controlled by a network of genes rather than by a single master regulator gene [101]. In this context, complex interactions among genes that respond to both internal and external stimuli play a key role in determining economically important production traits in livestock, rather than any one master gene. Advanced technologies such as RNA-seq, qPCR, and microarrays allow for comprehensive gene expression profiling and targeted gene analysis. Additionally, spatialomics enables spatially resolved gene expression at the single-cell level, which provides insights into complex tissue interactions. CRISPR-Cas9 offers precision genome-editing capabilities, targeting specific genes like ATP1A1 associated with heat resilience. Further, pathway analysis tools like the KEGG database and COG analysis support the exploration of metabolic and stress-response genes, identifying key biological markers for thermoresilience.

The next challenge in utilizing insights from these technologies is to enhance livestock production systems by identifying animals with desirable traits and integrating them into precise breeding programs or management strategies.

## 12. Future Perspectives

Recently, most of the transcriptomics-based studies were chosen for investigation because of their potential to boost productivity and enhance genetic quality, particularly in the livestock industry. However, spatialomics can be considered the next frontier to unravel complicated livestock traits and gene expressions. Spatialomics combines imaging, single-cell RNA sequencing, proteomics, and other types of analysis, allowing researchers to link between gene expression, protein expression, metabolomics, chromatin state, epigenomics, and other omics data to pinpoint the locations inside a tissue where specific transcripts are expressed and create highly complex images. Through this technology, the rate of genetic improvement can also be accelerated in the livestock sector, which can be more thermoresilient as well as productive in the face of future climatic fluctuations. Differentially expressed molecules identified from the analysis across multiple populations of livestock exposed to different environments will likely be more stout biological markers that can be implemented in future breeding policies.

## Figures and Tables

**Table 1:** Transcriptome analysis-identified genes associated with farm animal heat stress response.

Species	Genes Identified Through Transcriptome Analysis	Function	References
Bovine	Heat Shock 70 kDa Protein 1A (*HSPA1A*), Heat Shock 105 kDa/110 kDa Protein 1 (*HSPH1*), Heat Shock 70 kDa Protein 8 (*HSPA8*), DnaJ (*Hsp40*) homolog, subfamily A, member 1 (*DNAJA1*), and CDC-like kinase 1 (*CDK1*)	Metabolic process, catalytic activity, transcription factor activity, enzyme regulatory activity, protein binding, apoptotic process, stimulus response, cellular process, and immune system process	[35]
Bubaline	Heat Shock 70 kDa Protein (*HSPA6*), Heat Shock 22 kDa Protein (*HSPB8*), DnaJ (Hsp40) homolog, subfamily B, member 2 (*DNAJB2*), heat shock 70 kDa protein 1A (*HSPA1A*), MHC class I heavy chain (*BOLA*), mitochondrial ribosomal protein L55 (*MRPL55*), 6-phosphofructo-2-kinase/fructose-2,6-biphosphatase 3 (*PFKFB3*), proteasome (prosome, macropain) 26S subunit, ATPase, 2 (*PSMC2*), endonuclease domain-containing 1 (*ENDODD1*), AT rich interactive domain 5A (MRF1-like) (*ARID5A*), and SUMO1/sentrin/SMT3 specific peptidase 3 (*SENP3*)	Immune response, chaperon activity, cell proliferation, and metabolism	[36]
Ovine	*Rap1*, mitogen-activated protein kinase (*MAPK*), phosphatidylinositol 3-kinase and protein kinase B (*PI3K-AKt*), natriuretic peptide receptor 1 (*NPR1*), angiopoietin 2 (*ANGPT2*), and sodium-dependent citrate transporter member 5 (*SLC12A5*)	Fat regulation mechanism	[37]
Poultry	Heat Shock Proteins (*HSP*s), myosin light chain kinase 2 (*MYLK2*), and Bradykinin receptor B1 (*BDKRB1*)	Protein processing and synthesis	[38]
Swine	Solute carrier family 16 member 2 (*SLC16A2*), membrane-associated ring-CH-type finger 1 (*MARCHF1*), TNF alpha-induced protein 6 (*TNFAIP6*), relaxin/insulin-like family peptide receptor 2 (*RXFP2*), and interleukin 15 (*IL15*)	Heat resistance, inflammatory response, and immune function activation	[40]

**Table 2:** Molecular biomarkers for thermoresilience in various farm animals.

Gene	Description	Function	Animals	References
*HSP27/HSPB1*	Heat shock protein 27	Protects cells from cytotoxic effects of protein misfold	Cattle, Goat, Buffalo	[40,78,79,80,81,82]
*HSP40/DNAJB1*	Heat shock protein 40	Stimulate ATPase activity of Hsp70.		
*HSP60/HSPD1*	Heat shock protein 60	Transportation and refolding of proteins		
*HSP70/HSPA1A*	Heat shock protein 70	For protein folding and help to protect cells from stress.		
*HSP90AB1*	Heat shock protein 90	Signal transduction, protein folding, Protein degradation, and morphologic evolution		
*HSF1*	Heat Shock Factor 1	Primary mediator of transcriptional responses to proteotoxic stress with important roles in non-stress regulation such as development and metabolism.	Goat, Cattle	[77,83]
*ACACA*	Acetyl-CoA carboxylase alpha	Fatty acid metabolism; Lipoprotein lipase (LPL) is the enzyme in milk responsible for enzymatic lipolysis, i.e., the hydrolysis of fatty acids from triglycerides and phospholipids in the milk	Cattle	[74,84,85]
*FASN*	Fatty acid synthase			
*LPL*	Lipoprotein lipase			
*TLR2*	Toll-like receptor 2	Toll-like receptor 2 (TLR 2) and Toll-like receptor 4 (TLR 4) recognize the damage-associated molecular patterns (DAMPs) to produce several pro-inflammatory cytokines to evoke the host immune response during heat stress	Cattle	[19,86,87]
*TLR4*	Toll-like receptor 4			
*DUSP1*	Dual specificity protein phosphatase 1	Regulates MAPKs activity and play an important role in the cellular response to environmental stress.	Buffalo, Cattle, Sheep, and Goat	[40,88,89]
*GPX1*	Glutathione peroxidase 1	One of the most important antioxidant enzymes, functions in detoxification of hydrogen peroxide	Buffalo, Cattle	[40,90,91]
*ATP1A1*	Sodium–Potassium Adenosine Triphosphatase	Transport of sodium and potassium ions across cell membranes	Buffalo, Cattle	[92,93,94]
*TNFA*	Tumor necrosis factor	Immune responsive genes	Goat, Buffalo, Rabbit	[40,84,95]
*IL6*	Interleukin 6			
*IL8/CXCL8*	Interleukin 8			
*NF-kβ*	Nuclear factor kappalight- chain enhancer of activated B cells			

## Data Availability

No new data were created or analyzed in this study.
